# After the Storm: Regeneration, Repair, and Reestablishment of Homeostasis Between the Alveolar Epithelium and Innate Immune System Following Viral Lung Injury

**DOI:** 10.1146/annurev-pathmechdis-031621-024344

**Published:** 2022-10-21

**Authors:** Joseph D. Planer, Edward E. Morrisey

**Affiliations:** 1 Department of Medicine, Perelman School of Medicine, University of Pennsylvania, Philadelphia, Pennsylvania, USA; 2 Penn-CHOP Lung Biology Institute, Perelman School of Medicine, University of Pennsylvania, Philadelphia, Pennsylvania, USA; 3 Penn Cardiovascular Institute, University of Pennsylvania, Philadelphia, Pennsylvania, USA; 4 Department of Cell and Developmental Biology, Perelman School of Medicine, University of Pennsylvania, Philadelphia, Pennsylvania, USA

**Keywords:** alveolar epithelial cell, influenza, repair, regeneration, immune-epithelial interactions

## Abstract

The mammalian lung has an enormous environmental-epithelial interface that is optimized to accomplish the principal function of the respiratory system, gas exchange. One consequence of evolving such a large surface area is that the lung epithelium is continuously exposed to toxins, irritants, and pathogens. Maintaining homeostasis in this environment requires a delicate balance of cellular signaling between the epithelium and innate immune system. Following injury, the epithelium can be either fully regenerated in form and function or repaired by forming dysplastic scar tissue. In this review, we describe the major mechanisms of damage, regeneration, and repair within the alveolar niche where gas exchange occurs. With a focus on viral infection, we summarize recent work that has established how epithelial proliferation is arrested during infection and how the innate immune system guides its reconstitution during recovery. The consequences of these processes going awry are also considered, with an emphasis on how this will impact postpandemic pulmonary biology and medicine.

## 1. INTRODUCTION

Mammalian physiology depends on the proper functioning of a respiratory system that serves a number of functions, most importantly gas exchange. To accomplish this, the mammalian lung has evolved an intricate architecture of branching airways and alveolar parenchyma that both surveilles the exposed barrier to the external environment and maximizes the surface area for gas exchange. A natural consequence of these functions is that the lung is especially sensitive to infectious and noninfectious environmental insults.

Over the last several decades, foundational work in immunology and microbiology has allowed us to understand the lung as an arena for interactions between various infectious microbes and the host’s innate and adaptive immune defenses. The comparative paucity of techniques for studying repair and regeneration in the lung has meant that characterization of lung injury patterns and mechanisms of repair and regeneration have lagged substantially. Nevertheless, advances in generation of genetic mouse models and DNA sequencing technology over the last several decades have fostered the creation of new tools and facilitated studies of the developmental and regenerative biology of the lung. These recent studies have given us unprecedented insight into the processes by which lung function is fully restored after acute injury, which we refer to as regeneration, and by which lung healing occurs with scar formation, which we refer to as repair.

In this review, we focus our discussion on the mechanisms of repair and regeneration in the lung’s alveolar epithelium following acute lung injury, with an emphasis on the response to viral infection. Reviews on the pathogenesis of acute lung injury and the host immune response to infection or insult have been published recently, and we refer readers to those reviews for further discussions of these subjects (1–3).

## 2. THE SUSCEPTIBLE EPITHELIUM OF THE RESPIRATORY TRACT

The respiratory system can be separated into multiple anatomic compartments on the basis of tissue structure and function and cellular composition. To discuss the repair and regeneration of lung architecture, we briefly review the major niches that can serve as sites of infection before focusing on how epithelia and immune cells act in conjunction to restore homeostasis in the injured alveolus. We note that several recent reviews have comprehensively addressed the composition of the anatomic and cellular compartments of the mammalian lung (4–6).

### 2.1. Upper Airway

The upper airway consists of structures proximal to the vocal cords, including the nasal passages and nasopharynx, oropharynx, and larynx. While these structures have important nonrespiratory functions (e.g., phonation and olfaction), they also perform important roles in respiration including humidifying ambient air, entrapping large particulate matter, and priming mucosal immune responses. Despite myriad host defenses such as production of mucus, secreted immunoglobulins, and antimicrobial peptides, the upper respiratory tract is the most common site of respiratory viral infections (7). Although these epithelia are distinct from the alveolar epithelium in both form and function, they serve as the first site of encounter for pathogens and may amplify viruses that ultimately cause greater damage in the lower airways and alveolar epithelium.

### 2.2. Lower Conducting Airways

The lower airways include regions of the respiratory tract below the level of the vocal folds that do not participate in gas exchange but play distinct roles in respiratory physiology. These structures, which include the trachea, bronchi, and bronchioles, are lined by a pseudostratified columnar epithelium, interspersed with a self-renewing population of basal cells, ciliated cells, goblet cells, neuroendocrine cells, secretory cells, and recently described ionocytes (reviewed in 8). In addition to serving as a conduit for air, intrathoracic airway epithelium also actively clears mucus, environmental particles, and microbes from the lower passages via rhythmic beating of cilia. Lower airway epithelium also serves as a principal site of viral infection in both humans and mice, and a large body of literature has characterized the roles of viruses in causing exacerbations of chronic pulmonary diseases such as asthma and chronic obstructive pulmonary disease (COPD) (9, 10).

### 2.3. Respiratory Airways

The respiratory airways lie distal to the bronchioles and represent a unique structure that is not fully conserved among mammals. In humans, the distal airways give rise to terminal bronchioles, which in turn open into respiratory bronchioles (RBs). RBs are delineated histologically and functionally by the presence of interspersed alveoli as well as a transition to low cuboidal epithelium. This anatomic niche does not exist in the mouse airway, which terminates at the bronchoalveolar duct junction (BADJ) without a corresponding structure containing both airways and alveoli. Recently, a new cell type was identified in the RB, termed the respiratory airway secretory cell, which was found to act as a progenitor for alveolar epithelial cells (11). Thus, this region of the human lung, which is absent in mice, may serve as an important niche for the regenerative response after acute injury. A population of cells found at the BADJ and termed bronchioalveolar stem cells have been reported in mice and have been reported to differentiate into alveolar epithelial cells as well (12). The respiratory airways are a common site of viral lung infection, particularly in children where infectious bronchiolitis has been associated with long-term respiratory dysfunction (13).

### 2.4. Alveolar Epithelium

**Figure 1:**
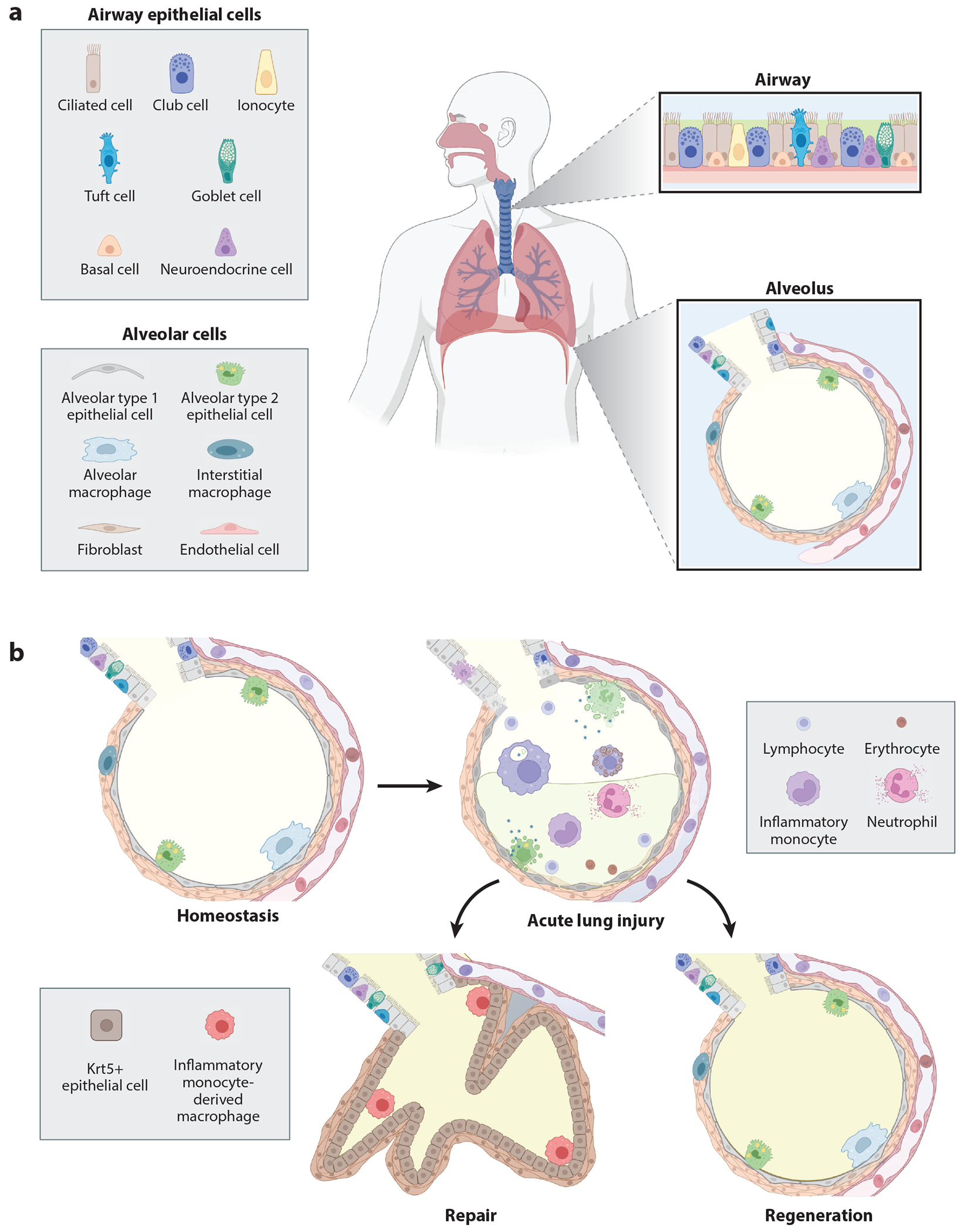
(*a*) Epithelium of the lower airway and composition of the alveolus. (*b*) Repair and regeneration of the alveolus following injury. At homeostasis, the alveolar epithelium consists of squamous alveolar type 1 epithelial cells that are located in close contact with the capillary bed to facilitate gas exchange, as well as cuboidal alveolar type 2 epithelial cells that secrete surfactant stored in lamellar bodies. The alveolus is surrounded by a sparse interstitium composed of fibroblasts, interstitial macrophages, and other cell types not depicted here (e.g., lymphatic vessels and nerves). Viral injury causes alveolar epithelial cell death, barrier dysfunction, impaired gas exchange, alveolar hemorrhage, and infiltration of leukocytes and protein-rich fluid. Resolution of inflammation can occur via regeneration (reconstitution of the functional alveolus) or repair (scarring). Repaired epithelium does not participate in gas exchange and contains airway-derived cuboidal epithelial cells expressing Krt5+. Abbreviation: Krt5, keratin 5. Figure adapted from images created with BioRender.

The alveolus is the functional unit of the lung and the principal site of gas exchange. The alveolar epithelium consists principally of alveolar type 1 (AT1) and alveolar type 2 (AT2) cells. AT1 cells are specialized squamous cells that cover the large majority of the alveolar surface area and are closely apposed to the capillary network (Figure 1). In contrast, AT2 cells are cuboidal cells that produce and recycle the surfactant proteins and phospholipids that maintain surface tension within the alveolus, preventing injury from expansion and collapse during the respiratory cycle (14). Recent advances in single-cell RNA sequencing (scRNA-seq) have yielded much deeper insight into the cellular heterogeneity of the alveolar epithelium (6, 15, 16). During injury, AT2s also function as an important progenitor cell population that can reconstitute the alveolar epithelium by both proliferating and differentiating into AT1s (see below for further discussion). Since AT2 cells are also important targets of viral infections, how alveolar repair can proceed following their loss remains an area of active research and debate.

### 2.5. Immune System

The enormous surface area of the respiratory tract necessitates a broad range of host defense mechanisms to control and clear pathogens, recycle cell debris and surfactant, and sequester noxious particles. These immune functions are carried out by nonimmune cells [e.g., secretion of mucin and antimicrobial peptides by various epithelial lineages (17), propagation of the mucus layer proximally by ciliated epithelial cells], as well as by dedicated immune cells of hematopoietic and lymphopoietic origin. The cellular immune system consists of leukocytes that participate in innate and adaptive immune functions. Adaptive immune cells consist of B and T lymphocytes that reside within bronchus-associated lymphoid tissue aggregates that exist at homeostasis in some but not all species of mammals but can also develop in response to inflammation of infection (18). Additionally, intraepithelial T lymphocytes expressing either αβ or γδ T cell receptors are interdigitated within the airway epithelium and assist in defense and repair following injury (19). At homeostasis, innate immune cells compose a substantial fraction of the total cellular composition of the lung. These cells include dendritic cells, innate lymphoid cells, neutrophils, and multiple phenotypically distinct groups of monocytes and macrophages. Resident macrophage populations can be further subdivided into airway, alveolar, and interstitial macrophages. Alveolar macrophages are perhaps the most abundant and best-characterized innate immune cells in the lung and have a wide variety of functions both in homeostasis and in repair after injury (reviewed in 2, 3, and 20). They reside within the lumen of the alveolus and assist in surveilling for pathogens and maintaining surfactant homeostasis. In mice, they are derived from monocytes that are recruited from the fetal liver and yolk sac in multiple waves prior to birth (21, 22). After birth, their population is maintained by in situ proliferation (23) or recruitment of circulating blood monocytes during injury that subsequently acquire a transcriptional phenotype similar to resident alveolar macrophages over time (24). Less is known about how interstitial macrophages regulate alveolar homeostasis, although they have been postulated to play a largely immunoregulatory role (25).

### 2.6. Human Versus Mouse

Much of our understanding of the development, regeneration, and repair of the lung has been derived from transgenic murine models and studies of ex vivo human tissues. For many of the major cell types and structures in the lung, the mouse can serve as a reasonable model for human anatomy. At the cellular level, for example, mice and humans have similar alveolar epithelial cell lineages with many conserved transcriptional features between AT1s and AT2s. However, the dramatically different metabolic requirements and lifestyles of humans and mice have led to several important differences in their respective lung anatomy that need to be considered when comparing findings from studies in these organisms (reviewed in 26). For example, the large airways in humans have a dedicated bronchial circulation that is not present in mice, as well as cartilaginous rings that buttress airways for several generations distal to the trachea. As noted above, the airway to alveolar junction is highly divergent between mice and humans. These anatomic differences may also have consequences in viral lung disease, as viral infection of the distal airways in humans causes a substantial disease burden (27). For example, mice have substantially less pathology in response to cigarette smoke exposure, while ferrets and humans develop a distinct pathology of the RBs due to smoke injury (28).

## 3. ACUTE PULMONARY INJURY

The vast majority of cells in the lungs are quiescent at homeostasis, with a relatively modest rate of epithelial turnover and little organized immune activity. Due to the central location of the lungs in the circulatory system and the reliance on a thin epithelial barrier to accomplish gas exchange, the lungs are exquisitely sensitive to infectious, inflammatory, and mechanical insults. Acute lung injury develops in a short period of time (typically defined as under 1 week) and can be localized or diffuse. The following discussion focuses on acute infectious injuries, as these are the best-studied areas of repair and regeneration in humans and mice.

### 3.1. Respiratory Failure: The Acute Respiratory Distress Syndrome

Acute respiratory distress syndrome (ARDS) represents the severest manifestation of acute lung injury in humans and is accompanied by a substantial rate of mortality in spite of recent advances in treatment (29). ARDS is defined as an acute onset or worsening of respiratory symptoms, impaired oxygenation (manifest as hypoxemic respiratory failure), and bilateral lung infiltrates not explained fully by pulmonary edema from cardiac dysfunction (30). ARDS can arise from numerous disease processes, most commonly infectious and/or inflammatory. The initial description of ARDS pathology noted the presence of alveolar collapse, inflammation, and interstitial and intra-alveolar hemorrhage and edema, as well as hyaline alveolar membranes in nearly all patients (31). Since then, studies in humans and animal models have identified uncontrolled inflammation and the loss of alveolar epithelial barrier integrity as central mechanisms in the development of ARDS (32). The combination of inflammation and barrier dysfunction leads to filling of alveolar spaces with protein-rich exudative fluid and inflammatory leukocytes that help to control infection but can also propagate tissue damage (Figure 1). A subset of patients who experience sustained severe injury eventually develop a second pathologic phase of disease (termed the fibroproliferative phase) characterized by persistent inflammation, aberrant reepithelialization, and expansion of fibroblasts with excessive deposition of extracellular matrix (33). While many patients who survive eventually return to their baseline lung function, a substantial minority experience residual loss of both mechanical lung function and gas exchange (alveolar) interface (34).

While the concept of ARDS has some clinical utility in identifying patients with severe lung injury who benefit from protective ventilatory strategies, its focus on physiological parameters elides the multiple injury pathways that can lead to a common physiologic end point. As such, it serves mainly as a useful starting point for considering the various mechanisms of severe lung injury and repair.

### 3.2. Viral Lung Injury

The host immune response to viral lung injury has been studied extensively over the last half century and has been reviewed in depth recently (35, 36). Mammals have evolved a wide array of defenses to limit damage from viral replication and immune injury that range from cell-intrinsic viral sensors to complex cell signaling networks. Although a full discussion of these topics is beyond the scope of this review, we briefly summarize some key features of the host response to viral lung infection below, focusing on the biology of the epithelial cell compartment in two injury models.

#### 3.2.1. Influenza.

Influenza causes substantial morbidity and mortality through yearly cycles of endemic transmission as well as in epidemics that follow the emergence of new strains (37). Influenza A virus (IAV), the most common type, infects airway and respiratory epithelia, leading to disease through both direct damage to the epithelium and inflammation. Given its human relevance and amenability to study in rodents, IAV has become the predominant model for studying viral lung injury.

Influenza is transmitted from person to person via inhalation of droplets produced during coughing, sneezing, or talking. IAV virions bind to α-2,3 and α-2,6 sialylated glycans on the surface of host epithelia, with individual strains of IAV exhibiting tropism for epithelial cells at different points along the full length of the lower respiratory tree (38, 39). Tissue tropism for a given strain of IAV also varies between mammals (40), reinforcing the importance of considering assumptions about host features in animal models of influenza. Significant heterogeneity exists in the severity of murine infection as a function of both influenza strain and host genetic background (41, 42). This has both complicated the interpretation of discordant experimental results and yielded insights into important features of host-viral interactions, such as the identification of cellular proteins such as Mx1 that help to restrict influenza replication (42, 43). For the purposes of this review, we focus on viruses with tropism for the lower respiratory tract, where IAV has been reported to infect bronchial and bronchiolar epithelial cells, AT1s and AT2s, and alveolar macrophages (39, 40).

Viral binding to epithelial cells is mediated by the interaction between the viral hemagglutinin protein and host cell-surface sialic acid residues. Following binding, the virus is internalized via the endosomal pathway, with cytosolic release occurring after acidification of the endosome. Viral RNA is released into the host cytosol and imported into the nucleus where viral replication occurs, followed by virion assembly in the cytoplasm, budding, and release (44). Epithelial cell death is common and can occur via apoptosis or necrosis (45).

At the cellular level, hosts have evolved multiple lines of defense against viral infections that limit infectivity and inflammation due to IAV. Innate immune sensing in target epithelial cells represents the first line of defense and consists of cell-intrinsic proteins that sense and respond to the presence of pathogen-associated molecular patterns. These pathogen recognition receptors (PRRs) include multiple Toll-like receptors (TLRs), retinoic acid–induced gene-I protein (RIG-I), and NLR family pyrin domain containing 3 (NLRP3) (36). Of these, TLR3, RIG-I, and NLRP3 are expressed in bronchial and/or alveolar epithelia and have been implicated in the downstream activation of innate immune pathways (46). Binding of viral RNA to intracellular PRRs activates signaling through interferon response factors 3 and/or 7, as well as nuclear factor kappa B, leading to transcription of interferons as well as proinflammatory cytokines such as interleukins 1 and 6 (IL-1 and IL-6) and tumor necrosis factor α (TNF-α). Activation of these responses thereby leads to infiltration of both innate and adaptive immune cells, resulting in a coordinated antiviral response. In mice, viral loads in the lung peak within the first week (41, 47) and interferon responses return to baseline shortly thereafter (48). Epithelial repair and regeneration crescendo during the second week after infection and can take several weeks to complete.

#### 3.2.2. Coronaviruses.

Prior to the coronavirus disease 2019 (COVID-19) pandemic, coronaviruses were mostly encountered as upper respiratory pathogens, typically causing mild seasonal infections. Two earlier outbreaks, severe acute respiratory syndrome coronavirus (SARS-CoV) in 2002 and Middle East respiratory syndrome coronavirus (MERS-CoV) in 2012, marked the first recorded emergence of coronaviruses with tropism for the alveolar epithelium, resulting in increased morbidity. Fortuitously, these strains circulated widely enough to raise alarm without causing global pandemics, thus permitting the scientific community to establish a basic understanding of their biology prior to the outbreak of SARS-CoV-2 (reviewed in depth in 49). Both SARS-CoV and SARS-CoV-2 bind to angiotensin-converting enzyme 2 (ACE2), which acts as the cell-surface receptor in conjunction with the transmembrane serine protease 2 (50, 51). ACE2 is expressed broadly in multiple different tissues. In the lungs, it is expressed at highest levels on AT2 cells and alveolar macrophages, which have been proposed to be the principal cells responsible for causing pulmonary pathology (52, 53). Transcriptional surveys of patients with fatal COVID-19 suggested that macrophage activation, impaired alveolar epithelial regeneration, and pathologic fibroblast activation are hallmarks of severe pulmonary disease (54). Studies in the explanted lungs of patients who received lung transplantation revealed widespread tissue destruction and the complete loss of normal architecture, and replacement with connective tissue, cuboidal epithelium, and hemosiderin-laden macrophages (55). Transcriptional profiling in these patients also demonstrated an abnormal epithelial response with expansion of a transitional AT2 cell state (see below) and overrepresentation of inflammatory monocytes relative to healthy tissue. These experiments have also been corroborated in studies using cultured human AT2 organoids that demonstrate robust infection with SARS-CoV-2 as well as induction of interferon responses and epithelial growth arrest (56).

## 4. THE AIRWAY-DERIVED DYSPLASTIC RESPONSE TO VIRAL LUNG INJURY

Like most vital organs, the mammalian lung reacts to injuries of varying severity with responses that can both limit the extent of destruction and facilitate repair. However, analogous to scarring in other organs, the lung can also respond to severe injury by sacrificing functional tissue to limit further damage. This process of dysplastic alveolar repair has recently been reviewed in depth (57).

### 4.1. Historical Descriptions

Early reports of an aberrant epithelial response to severe viral lung injury described a morphologically distinct epithelial cell population that was present in the distal airways of patients who died following infection with H2N2 influenza (58). These dysplastic epithelial pods were predominantly located in the peribronchiolar region, suggesting the possibility of a relationship with the airway epithelium. They were subsequently found to express keratin 5 (Krt5) and were noted to emanate from a transformation related protein 63 (Trp63)-expressing lineage of epithelial cells in the distal airways (59). Subsequently the progenitor cell population giving rise to these Krt5 pods was termed either distal airway stem cells (60) or lineage-negative epithelial progenitors (LNEPs) (61).

### 4.2. Origin and Composition of Krt5 Pods

The cell type of origin for Krt5 pods has been extensively researched and debated. Initial descriptions of Krt5+ cells indicated that they arose from rare basal cell–like progenitors in the distal intrapulmonary airways rather than the trachea (59, 61). At homeostasis, these cells express p63 but not Krt5, and they later acquire Krt5 expression during injury (61, 62). The early characterization of Krt5 pods suggested that they could generate mature AT1 and AT2 cells (59). However, subsequent studies using fate-mapping approaches have demonstrated that regeneration of the alveolar epithelial lineages by airway cells expressing Krt5 does not occur at a substantial frequency following influenza infection (reviewed comprehensively in 57).

Dysplastic remodeling with Krt5-expressing cells is also observed following viral infection in humans, suggesting that this process is evolutionarily conserved within mammals. Pathologic descriptions of a phenomenon of squamous metaplasia have been reported in cases of severe injury with diffuse alveolar damage (63). Subsequent work demonstrated Krt5 staining in these peribronchiolar pods in cases of idiopathic acute lung injury with ARDS (64), idiopathic pulmonary fibrosis (61), influenza (65), and COVID-19 (66). As in mice, these cuboidal epithelial cells do not express the canonical alveolar epithelial cell markers, indicating that they do not successfully reconstitute the functional alveolus (64, 67). The conservation of a generalized epithelial dysplastic response to injury across mammals suggests an important evolutionary role in either constraining damage, maintaining barrier function, or epithelial regeneration.

### 4.3. Cellular Signaling of Dysplastic Epithelialization

Recent work has improved our understanding of the cell signaling processes that promote dysplastic epithelialization after injury. The stimulus to generate bronchiolization with Krt5 pods seems to derive partially from a loss of AT2 cells in conjunction with secondary signals. Using a model in which diphtheria toxin expression can be induced in AT2 cells, resulting in their ablation, Yee et al. (68) demonstrated that AT2 cell loss alone was not sufficient to induce Krt5+ cell accumulation. Work by Xi et al. (65) using a model of H1N1 IAV demonstrated that the accumulation of Krt5 pods occurred in response to local hypoxia and was dependent on expression of hypoxia inducible factor 1α (HIF1α), as deletion of HIF1α led to nearly complete loss of peribronchial Krt5 pods. Interestingly, HIF1α^−/−^ mice had less severe disease as evidenced by milder hypoxemia, less pulmonary edema, and a higher proportion of AT2 cells in the epithelium. The authors subsequently demonstrated that hypoxia induced Notch signaling, which favored the expansion of Krt5+ cells. In contrast, signaling via the Wnt/β-catenin pathway promoted differentiation of LNEPs into AT2s, indicating that local signaling cues (rather than prior cell fate commitment) dictated the path of differentiation (65). Intriguingly, it was recently reported that hypoxia promotes differentiation of tracheal basal cells toward solitary neuroendocrine cell fate in a HIF1α/HIF2α-dependent manner (69). Expansion of these neuroendocrine cells helped to protect from hypoxic injury via secretion of calcitonin-related gene peptide. Together, these studies indicate that hypoxia can induce different cellular responses from the airways, with additional signaling or anatomic cues likely determining how airway epithelia respond.

Fibroblast growth factors (Fgfs) also play important roles in promoting alveolar epithelial growth and differentiation (70–73). A recent study by Yuan et al. (72) implicated the Fgf10-Fgfr2b signaling in the fate choice between Krt5 and AT2. After observing that Fgf10+ fibroblasts were found adjacent to AT2 cells, the authors used a combination of inducible knockouts and fate mapping to demonstrate that loss of Fgfr2b signaling led to loss of AT2 and Krt5+ cells following bleomycin injury (72). Overexpression of Fgf10 in bronchial epithelial cells, conversely, promoted the accumulation of AT2 cells. Together, these data indicate that Fgf signaling plays an important role in promoting differentiation of airway basal cells to repopulate the alveolar space with either AT2s or dysplastic epithelial cells. Additional studies are needed to determine the local cues that govern regulation of the Fgf pathway.

### 4.4. Persistence of Pods and Long-Term Effects on Respiratory Function

The dysplastic epithelial response results in the replacement of previously functional alveolar tissue with bronchiolized epithelium that does not apparently participate in gas exchange. This can result in worsened hypoxemia (65) and is correlated with delayed viral clearance and prolonged inflammation (74). One major consequence of the COVID-19 pandemic has been the dramatic increase in the incidence of ARDS and the recognition of limitations in exercise tolerance (75), impairments in gas exchange (76), and persistent chest imaging abnormalities (77) that last for months or longer after viral infection. While Krt5+ cells have been identified in scRNA-seq data sets (78) and pathology specimens (66) from patients with severe COVID-19, more work is needed to determine whether our current understanding of the dysplastic epithelial response to injury is generalizable between different types of viral injury.

## 5. EPITHELIAL REGENERATION

The advent of scRNA-seq has allowed an unprecedented view into the composition of the alveolus at a cellular and transcriptional level (6, 79). While these studies have permitted us to define some of the cross talk between cellular compartments (80), more work needs to be done to understand the timing and context dependency of these signaling networks during epithelial repair and regeneration following viral injury.

### 5.1. AT2 Cells as Niche Progenitors

**Figure 2:**
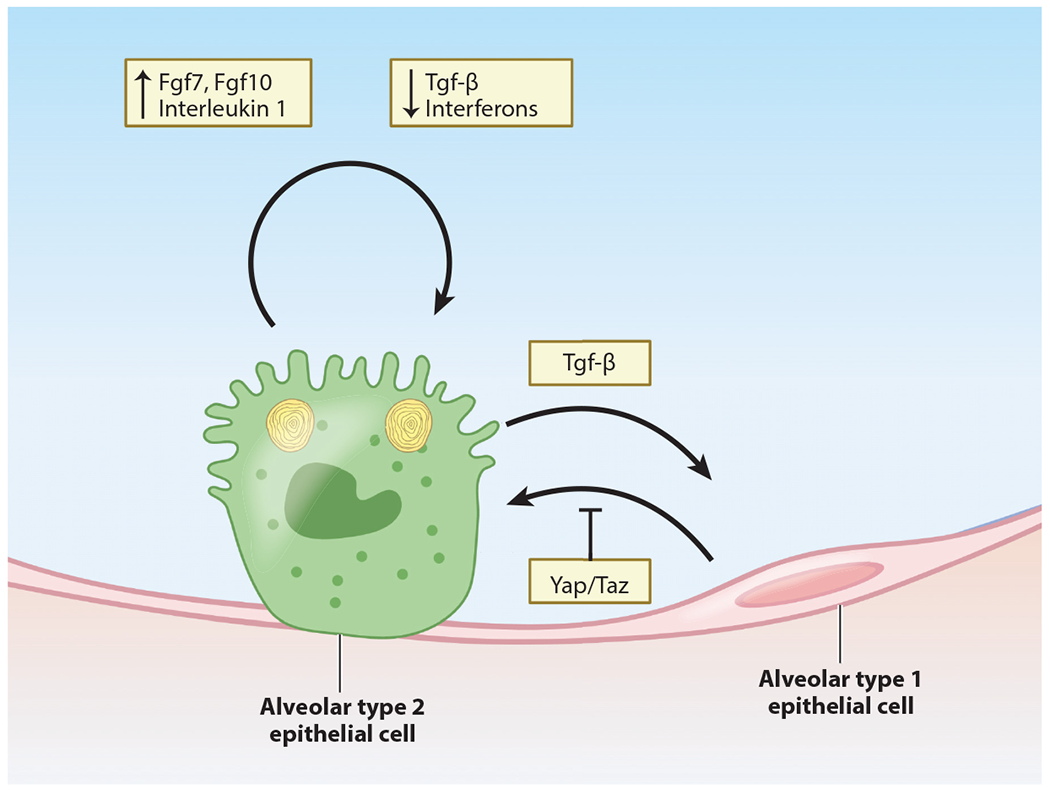
Alveolus intrinsic signals that promote proliferation and differentiation. AT2 cells serve as facultative progenitors for the alveolar epithelium. Recent work has uncovered subpopulations of AT2 cells that are preferentially endowed with proliferative capacity. AT2 cells are capable of regenerating both AT2 and AT1 lineages via proliferation and differentiation, respectively. Recent work has identified extracellular signals that promote (fibroblast growth factors, interleukin 1) or restrict (Tgf-β, interferons) AT2 cell proliferation. In contrast, Tgf-β promotes differentiation of AT2 cells into AT1 cells, while intracellular Yap/Taz signaling actively maintains AT1 identity by preventing differentiation back to the AT2 lineage. Abbreviations: AT1, alveolar type 1; AT2, alveolar type 2; Taz, transcriptional coactivator with PDZ-binding motif; Tgf-β, transforming growth factor beta; Yap, Yes-associated protein. Figure adapted from images created with BioRender.

AT2s have been identified as playing a central role in regenerating the damaged or denuded alveolus for several decades (81, 82). While the uninjured alveolus is quiescent during homeostasis in adults, AT2 cells reenter the cell cycle after most injuries and rapidly differentiate into AT1 cells (82). A recent report revealed that the ability of AT2 cells to differentiate into AT1 cells after hyperoxic lung injury is restricted to the adult lung and does not occur in the neonatal lung, suggesting differences in AT2-AT1 differentiation across the life span (83). Whether such differences are observed after infectious injury such as influenza remains to be explored. Despite these age-dependent differences, the AT2 cell remains the best-characterized alveolar epithelial progenitor in the adult lung in both humans and mice (Figure 2).

As with distal airway epithelial progenitor cells, investigation into AT2 cell heterogeneity has revealed distinct subsets that are endowed with progenitor capacity. Two recent studies reported the existence of a subpopulation of AT2s that expressed Axin2, a marker of Wnt responsiveness, and that served as alveolar epithelial precursors (73, 84). These cells, termed alveolar epithelial progenitors (AEPs), constituted a minority of AT2s at homeostasis and were capable of both proliferating and differentiating into AT1s following influenza infection. Another study showed that AT2 cells expressing the IL-1 receptor had an enhanced ability to proliferate and differentiate into AT1 cells (15). The growing number of scRNA-seq data sets has suggested additional AT2 heterogeneity, but most of these subpopulations have not been rigorously characterized for functional differences.

Recent studies have begun to identify and characterize the molecular pathways that regulate AT2 cell self-renewal and differentiation into AT1 cells. As noted above, the Axin2+ AEP subpopulation is preferentially responsive to Wnt signaling, which promotes AT2 self-renewal and fate while inhibiting AT1 differentiation (70, 73, 84). Wnt signaling can also be activated in AEPs by inhibition of lymphotoxin beta receptor (85). Fibroblast growth factors expressed in the alveolar niche also provide important signaling cues that guide alveolar epithelial growth, differentiation, and fate maintenance. Wnt-responsive AEPs were also characterized by their increased sensitivity to Fgf signaling via Fgf7 and Fgf10 in organoid culture compared with non-AEP AT2s. More recently, several studies have shown that Fgfr2 signaling is critical for the AT2 cell proliferative response to acute injury (71, 86, 87). Loss of Fgfr2 in AT2 cells also enhances their differentiation into AT1 cells, indicating that Fgf signaling helps to maintain the AT2 cell fate in response to injury (87).

The Hippo signaling pathway plays key roles in stem/progenitor cell self-renewal and cellular mechanotransduction (88, 89). The transcriptional effectors Yap and Taz are located in the cytoplasm when Hippo is active and translocate into the nucleus when Hippo signaling is inhibited, where they bind to TEAD transcription factors to regulate gene expression. Inhibition of Hippo signaling, either through expression of activated Yap/Taz mutants or loss of important Hippo kinases such as Lats2 or Mst1/2, leads to precocious activation of AT1 marker genes during lung epithelial development (90). In the adult lung, Yap expression has been shown to promote AT2 cell proliferation and inhibit AT1 differentiation in infectious models of lung injury (91).

The transforming growth factor beta (Tgf-β) superfamily also plays an important role in AT2 self-renewal and AT1 differentiation. Several reports have shown that high levels of bone morphogenetic protein (Bmp) signaling in the adult AT2 cell population promote AT2-AT1 differentiation whereas inhibition of Bmp signaling promotes AT2 self-renewal (92). While the role for cell autonomous Tgf-β signaling in adult AT2 cells after injury remains unclear, many organoid models used to study AT2 cells include Tgf-β inhibitors, which increase AT2 cell growth in these conditions (87, 93).

### 5.2. AT1-AT2 Plasticity During Alveolar Regeneration

While AT2-AT1 differentiation is a cardinal feature of alveolar regeneration in the adult lung, there is emerging evidence that AT1 cells can reprogram back into AT2 cells after certain lung injuries. To date, there is little evidence that AT1 cells can proliferate. However, Jain et al. (94) showed that a small number of AT1 cells can reprogram back into AT2 cells following pneumonectomy. More recently, Penkala et al. (83) showed that AT1-AT2 reprogramming was robust after hyperoxic injury in the neonatal and adult lung. This is in contrast to the limited ability of AT2-AT1 differentiation, which is restricted to the adult lung and not to the neonatal lung. Characterization of this reprogramming process identified Hippo signaling as an AT1-restricted pathway and showed that loss of Yap and Taz leads to a spontaneous reprogramming of AT1 cells into AT2 cells. These studies reveal the extensive nature of alveolar epithelial cell plasticity, how it changes across the life span, and how it modulates tissue regeneration after acute injury in the lung. Notably, the above findings derive largely from murine studies, although advances in culturing primary human alveolar epithelial cells will likely yield additional insights into the role of Hippo and other cell signaling pathways in human alveolar epithelial fate maintenance.

### 5.3. Intermediate States in AT2-AT1 Differentiation

Recent studies have identified an intermediate or transitional state that exists between AT2 and AT1 cells after acute injury. Using various methods including scRNA-seq and lineage tracing, these studies show that there is a subpopulation of transitioning cells that express high levels of markers such as claudin 4 and Krt8 (15, 16, 95, 96). Under normal situations, these transition state cells will ultimately become mature AT1 cells. However, it remains unclear whether cells could become blocked at this state in certain lung injuries or diseases. Further studies will be required to more fully assess the functional importance of this state during normal repair and in acute and chronic lung disease.

## 6. ALVEOLAR EPITHELIAL RESPONSES TO IMMUNE SIGNALING

**Figure 3:**
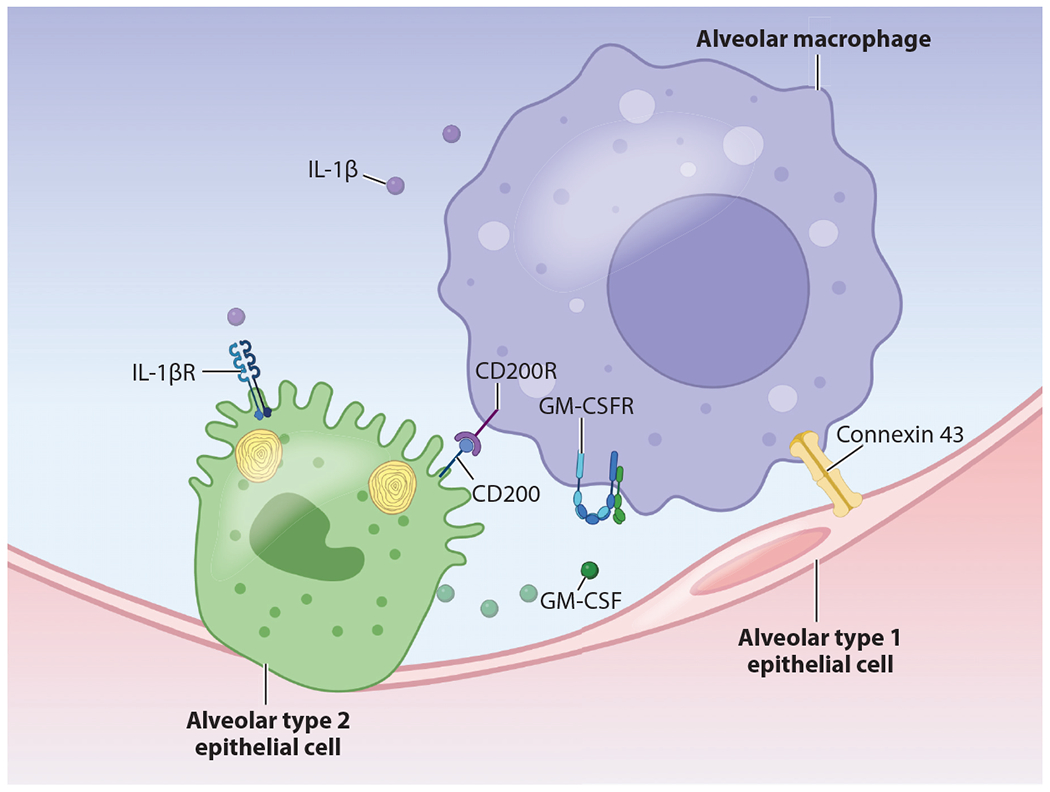
Immune signaling in the alveolus. At homeostasis, the alveolus is maintained in a quiescent state via bidirectional signaling between the alveolar epithelium and alveolar macrophages. These mechanisms include production of soluble factors such as IL-1, which is secreted by myeloid immune cells and promotes AT2 cell proliferation via the IL-1 receptor. GM-CSF is secreted by AT2 cells and signals via PPAR-γ to induce a transcriptional program that promotes surfactant homeostasis. AT2s also express the cell surface ligand CD200, which provides immunoregulatory signals to alveolar macrophages via CD200R. Several recent studies have also shown coordinated calcium-mediated signaling via gap junctions (notably connexin 43). Abbreviations: AT2, alveolar type 2; GM-CSF, granulocyte-macrophage colony-stimulating factor; IL, interleukin; PPAR, peroxisome proliferator-activated receptor. Figure adapted from images created with BioRender.

Innate immune cells play central roles in protecting damaged epithelial surfaces and assisting in repair and regeneration in the skin, lung, and other environmental interfaces (97). Correspondingly, epithelial regenerative processes typically occur within an immune-biased milieu. Several studies conducted over the last decade have begun to disclose the importance of immune signaling pathways in both promoting and restricting regeneration and repair (Figure 3).

### 6.1. The Interferon Response

Interferons (IFNs) play a central role in protecting the host epithelium from the pathogenic effects of viral infection. Discovered in the late 1950s, interferon was first characterized as a soluble factor produced by the chick chorioallantoic membrane in response to influenza infection that could be isolated and used to inhibit the replication of both influenza and unrelated viruses in separate culture (98). Since then, three major classes of interferons have been identified: type I (IFN-α/IFN-β), type II (IFN-γ), and type III (IFN-λs) that vary in cell tropism and downstream effector function. Interferon signaling is accompanied by a dramatic reorientation of cellular behavior at the transcriptional, translational, and posttranslational level. This results in the upregulation of numerous interferon-stimulated genes (ISGs) and cessation of proliferation, protein synthesis, and other central aspects of host cellular biology.

As primary targets of viral respiratory infections, airway and alveolar epithelia play a critical role in early amplification of the interferon response. Shortly after infection with IAV, AT2s begin to secrete type I and III interferons in addition to other proinflammatory cytokines (99). Type I interferons signal through the interferon alpha receptor (IFNAR) that is expressed broadly in most tissues. In contrast to the IFNAR, the interferon lambda receptor is expressed predominantly in epithelial tissues including the respiratory epithelium (100, 101). Cultured AT2s that are infected with IAV or treated with exogenous interferon exhibit a dramatic transcriptional change with up- or downregulation of hundreds of genes (102). These genes included canonical ISGs such as *Isg15* (103) and *Mx1* that assist in viral control, as well as multiple chemokines that recruit immune cells (102). A full discussion of the effects of these pathways is beyond the scope of this review, as they have been well studied and reviewed elsewhere (103, 104).

The net consequence of interferon signaling on the lung epithelium is growth arrest. For example, AT2 organoids cultured in the presence of interferon are smaller, have decreased colony-forming efficiency (105), and have increased numbers of apoptotic cells (56). The mechanisms by which type I and III interferons exert these effects have been clarified substantially over the last several years. In a recent study, Major and colleagues (48) noted that very little epithelial proliferation occurred during the 5 days following murine H3N2 IAV infection, a period of time corresponding to substantial tissue injury and the height of interferon production. The disappearance of interferon protein corresponded to dramatically increased epithelial proliferation and differentiation, which ultimately abated during the second week after infection. Mice lacking type I and III interferon receptors on epithelial cells exhibited higher levels of epithelial proliferation, confirming the requirement for intact interferon signaling for growth arrest.

A complementary study by Katsura and colleagues (56) revealed similar findings in human AT2 cells infected with SARS-CoV-2. Viral infection led to a rapid upregulation of the expected type I and III interferons as well as their downstream targets and was associated with induction of markers of apoptosis, decreased numbers of proliferating Ki-67+ cells, and loss of canonical markers of AT2 identity. These findings correlated with transcriptional and histological findings in specimens obtained from humans with severe COVID-19 disease. Finally, the authors demonstrated that treatment of AT2 organoids with interferon reduced proliferation and increased apoptosis (56).

Together, these findings indicate that interferons can act as a double-edged sword, controlling viral infection in host alveolar epithelium but at the expense of alveolar cellular integrity and proliferative capacity. This likely serves as a brake on infection, as ongoing epithelial proliferation would simply provide a continuous source of susceptible host cells. The cessation of interferon signaling, however, results in an exuberant regenerative response that integrates other proinflammatory cytokines that are also produced in the injured alveolus.

### 6.2. Interleukins and Chemokines

Although many investigations of cytokine signaling have focused on how they influence immune behavior, several recent studies have highlighted important roles for inflammatory cytokines in promoting the growth of alveolar epithelial cells.

Recent work using AT2 organoids demonstrated that multiple cytokines that are produced during viral infection affect growth of AT2 cells in vitro (105). Notably, TNF-α, IL-1, and IL-17 increased the size of organoids by increasing epithelial cell proliferation. This finding was somewhat paradoxical considering that alveolar epithelial cells are not found in significant numbers in the regions of the lung with the densest immune infiltrates. To further explain this, the authors noted that AT2 proliferation was highest in the regions near damaged lung zones but only in mice with intact IL-1 signaling (105). Importantly, IL-1 exerted proproliferative effects both on AT2 cells and on the fibroblast cells used to support AT2s in the organoid assay, suggesting that multiple cellular constituents of the alveolar niche may respond additively or synergistically.

Subsequent work by Choi and colleagues (15) replicated the finding that IL-1β increased organoid size and implicated interstitial macrophages as a source of IL-1β that promoted this effect. Using scRNA-seq of in vitro organoids, the authors found that IL-1β induced a transcriptional response in AT2s that resembled intermediate states along an AT2 to AT1 differentiation axis observed in mice injured with bleomycin. The authors further noted that while sustained IL-1β signaling inhibited the full differentiation of AT2s into mature AT1s in culture, withdrawal of the cytokine from growth medium permitted differentiation to occur (15). Together, these findings demonstrate the importance of cytokine signaling in multiple cell types within the alveolar niche and suggest that alveolar regeneration may require the coordinated activities of multiple cellular compartments.

IL-10 plays an important role in restraining inflammation and promoting resolution of injury. IL-10 is constitutively present in both humans and mice (106) and is expressed by AT1s and AT2s (107) as well as interstitial macrophages (108). IL-10 signals via the suppressor of cytokine signaling 3 to suppress expression of inflammatory cytokines such as IL-1, IL-6, and TNF-α (109). The timing and regulation of IL-10 signaling are also important determinants of lung healing. For example, in a model of postinfluenza bacterial pneumonia, blocking IL-10 improved survival and decreased bacterial burden (110).

### 6.3. Surfactant

While surfactant has been traditionally recognized for its mechanical properties that prevent cyclic alveolar collapse during the respiratory cycle, the various protein components also have well-characterized immune functions. Regulation of the pulmonary surfactant pool is critically important at the cellular, tissue, and organismal levels. Additionally, IAV infection results in dysregulation of surfactant homeostasis, an outcome that may play a role in causing reduced lung compliance and the development of ARDS (111). Surfactant consists of four surfactant proteins (SP-A, -B, -C, and -D), secreted from specialized organelles in AT2 cells called lamellar bodies, as well as phospholipids, which compose 80–90% of surfactant by weight (reviewed in 14 and 112). While both AT2s and alveolar macrophages are capable of recycling surfactant (113), loss or dysfunction of alveolar macrophages leads to the pathologic accumulation of surfactant, resulting in pulmonary alveolar proteinosis in humans and mice (114). Elegant work in the 1990s in mice lacking granulocyte-macrophage colony-stimulating factor (GM-CSF) demonstrated an accumulation of lipoproteinaceous eosinophilic fluid in their lungs and increased susceptibility to pulmonary infections (115, 116). This phenotype depended on the expression of the GM-CSF receptor on alveolar macrophages, implicating their dysfunction in the failed clearance of surfactant (117). Further studies highlighted the role of peroxisome proliferator-activated receptor γ signaling downstream of GM-CSF (118, 119) as an important factor in regulating macrophage inflammation in the setting of IAV infection (120). AT2s represent the major source of GM-CSF production in the lung (121), suggesting that this signaling circuit plays a central role in maintaining alveolar homeostasis during and after viral infection.

SP-A and SP-D belong to a family of pattern recognition receptors called collectins that bind to foreign glycans and help to clear environmental particles and pathogens by binding to CD14 on cells of the monocyte-macrophage lineage (122, 123). SP-A and SP-D have been proposed to exert anti-inflammatory effects on alveolar macrophages through multiple mechanisms including preventing complement activation andTLR signaling (122, 124) and binding via signal-regulatory protein alpha (125, 126). Notably, SP-A and SP-D have also been shown to exert direct antiviral effects by binding to IAV hemagglutinin, further emphasizing the importance of restoring surfactant homeostasis following infection (127).

### 6.4. Cell–Cell Interactions Between the Epithelium and Innate Immune System

Homeostasis in the alveolar niche is maintained (and restored after infection) via bidirectional interactions between epithelial cells and innate immune cells. While some of these signals are transmitted via soluble signaling pathways, others depend on cell-to-cell contact. In aggregate, alveolar epithelial signaling through multiple pathways serves to prevent alveolar macrophage activation in the absence of inflammatory cues. Moreover, following inflammation, recruited cells of the monocyte-macrophage lineage persist in tissue and eventually adopt a transcriptional profile that is similar to native alveolar macrophages (24), suggesting that signals within the alveolar niche dictate the behavior of myeloid cells.

Alveolar epithelial cells exert a direct immunoregulatory effect on alveolar macrophages via interactions between CD200 and CD200R. AT2 cells express CD200, the ligand for CD200R that is expressed on alveolar macrophages (128, 129). Alveolar macrophages from mice lacking CD200 exhibit spontaneous secretion of IL-6 and TNF-α when cultured in the presence of lung epithelial cells. CD200 knockout also led to less severe influenza illness, pulmonary pathology, and inflammation, consistent with the finding in other models that monocytes and macrophages can cause inflammatory lung damage during IAV infection (129, 130).

In addition to its central role in epithelial growth and regeneration, Tgf-β also assists in maintaining homeostasis between the epithelium and innate immune system and returning to a quiescent state following injury. At homeostasis, Tgf-β is expressed by multiple cell types in the alveolar niche and is activated and stabilized in the extracellular environment by binding to integrin αVβ6 expressed on alveolar epithelial cells (131, 132). Yu and colleagues (131) demonstrated that expression of the Tgf-β receptor in CD11c+ myeloid cells (including alveolar macrophages) is required for the development and/or maintenance of alveolar macrophages. Mice lacking integrin αVβ6 develop spontaneous emphysema that can be abrogated by providing exogenous Tgf-β or knocking out the major alveolar matrix metalloproteinase Mmp12 (133). Notably, mice lacking integrin αVβ6 also have reduced mortality in response to multiple respiratory viral infections and exhibit greater type I interferon responses, although in contrast to other studies, increased levels of interferon did not correlate with susceptibility to postviral pneumococcal pneumonia (134). Together, these data indicate that Tgf-β promotes the maintenance of alveolar macrophages within the alveolar niche while restraining excessive inflammatory activation.

Recently, the gap junction protein connexin 43 (Cx43) was identified as a regulator of immune-epithelial interactions within the alveolus. Using an innovative ex vivo imaging approach, Westphalen et al. (135) demonstrated that spatially remote alveolar macrophages exhibit synchronized calcium spikes in culture when exposed to lipopolysaccharide (LPS). This phenotype was abrogated in macrophages lacking Cx43 and depended on direct communication via the alveolar epithelium. Loss of Cx43 in the alveolar epithelium resulted in increased inflammation in response to LPS challenge, suggesting that communication via Cx43 gap junctions may help to prevent excessive alveolar macrophage activation (135). These findings were subsequently validated in a human ex vivo culture system (136), although their physiologic relevance needs to be further clarified.

Cells of the monocyte-macrophage lineage can also promote epithelial growth in the absence of overt lung injury. Using a model of compensatory regrowth after pneumonectomy, Lechner and colleagues (137) observed an increase in interstitial and alveolar macrophages in the remaining lungs of mice that had recently undergone unilateral pneumonectomy. Consistent with the role of C-C motif chemokine ligand 2 (CCL2) in recruiting monocytes to sites of injury, the authors observed that these mice had increased levels of circulating CCL2. Mice lacking the receptor for CCL2 (CCR2^−/−^) had significantly less compensatory growth of the remaining lung, less proliferation of AT2 cells, and less differentiation of AT2 cells into AT1 cells (137). Notably, mice lacking CCR2 are also protected from influenza injury (130), so recruited monocytes likely play a multifaceted role in repair and regeneration that depends on the additional signaling cues within the alveolus.

The distal lung is an anatomic niche faced with the challenging reality of needing to protect a highly exposed surface from environmental insults and pathogens, while preventing unrestrained inflammation. This necessitates delicate balancing of interactions between alveolar epithelia and lung resident macrophages through multiple cell signaling pathways. Absent inflammatory cues, the alveolar epithelium is quiescent in adults and maintains an anti-inflammatory state in resident myeloid cells. During viral infection, the net effect of the integrated epithelial-macrophage response is to upregulate cell-intrinsic antiviral responses, recruit inflammatory leukocytes, and arrest epithelial proliferation. With successful clearance of virus and the cessation of interferon signaling, epithelial proliferation can occur and may in fact be promoted by the presence of other inflammatory cytokines such as IL-1. How these events are coordinated and ultimately completed remains an area of intense investigation.

## 7. FUTURE DIRECTIONS

Advances in lineage tracing and single-cell transcriptional profiling have begun to revolutionize our understanding of the developmental and regenerative biology of the alveolus. Over the last decade, we have dramatically expanded our understanding of the wealth of signaling inputs that restrain or promote alveolar epithelial growth both during development and following injury. In spite of these recent technological advances, no currently approved therapeutics promote or assist in lung regeneration. To reach a point where mechanistic biology can be translated into clinical therapies that restore lung function, we will need to further advance our understanding of the unique structural and signaling features within the alveolar niche.

Recently, we have gained insight into the immune signaling pathways required to maintain an anti-inflammatory state in the alveolus at homeostasis while permitting appropriate activation of immune cells in response to pathogens. From these studies, it is apparent that maintaining homeostasis is an active process that requires balanced signaling within the epithelial and immune compartments. The signaling mechanisms that guide alveolar epithelial cell fate maintenance remain partially described. For example, the environmental inputs that lead AT2 cells to undergo proliferation and differentiation into AT1s remain incompletely characterized.

The COVID-19 pandemic has confronted us with the deficiencies in our understanding of how the alveolus regenerates or repairs after viral injury. For example, it remains unclear what signals dictate whether an injured area of lung undergoes regeneration or repair. While the composition of immune cells in the damaged alveolus clearly changes over time, how these cells communicate with the reconstituting epithelium remains less certain. Detailed time-course experiments will be needed to clarify the cell signaling networks that are activated at various time points during regeneration. Given the heterogeneous nature of lung injury during viral infection, future studies will also need to account for spatial differences in cell signaling across the full range of tissue damage severity. While these studies will be labor intensive and will require computational expertise, they will hopefully offer an unprecedented view into the basic biology of the alveolus and the mechanisms that guide its restoration.
